# A State-of-the-Art Review on Advanced Joining Processes for Metal-Composite and Metal-Polymer Hybrid Structures

**DOI:** 10.3390/ma14081890

**Published:** 2021-04-10

**Authors:** Francesco Lambiase, Silvia Ilaria Scipioni, Chan-Joo Lee, Dae-Cheol Ko, Fengchao Liu

**Affiliations:** 1 Department of Industrial and Information Engineering and Economics, University of L’Aquila, Via G. Gronchi 18, Zona Industriale di Pile, 67100 AQ L’Aquila, Italy; silviailaria.scipioni@student.univaq.it; 2 Dongnam Regional Division, Korea Institute of Industrial Technology, Goryeong-gun 52845, Korea; cjlee80@kitech.re.kr; 3 Department of Nanomechatronics Engineering, Pusan National University, Busan 46241, Korea; dcko@pusan.ac.kr; 4 Department of Naval Architecture and Marine Engineering, University of Michigan, Ann Arbor, MI 48109, USA; liufc@umich.edu

**Keywords:** hybrid joints, advanced joining processes, polymer composites, riveting, fastening, welding, joining, bonding mechanism

## Abstract

Multi-materials of metal-polymer and metal-composite hybrid structures (MMHSs) are highly demanded in several fields including land, air and sea transportation, infrastructure construction, and healthcare. The adoption of MMHSs in transportation industries represents a pivotal opportunity to reduce the product’s weight without compromising structural performance. This enables a dramatic reduction in fuel consumption for vehicles driven by internal combustion engines as well as an increase in fuel efficiency for electric vehicles. The main challenge for manufacturing MMHSs lies in the lack of robust joining solutions. Conventional joining processes, e.g., mechanical fastening and adhesive bonding involve several issues. Several emerging technologies have been developed for MMHSs’ manufacturing. Different from recently published review articles where the focus is only on specific categories of joining processes, this review is aimed at providing a broader and systematic view of the emerging opportunities for hybrid thin-walled structure manufacturing. The present review paper discusses the main limitations of conventional joining processes and describes the joining mechanisms, the main differences, advantages, and limitations of new joining processes. Three reference clusters were identified: fast mechanical joining processes, thermomechanical interlocking processes, and thermomechanical joining processes. This new classification is aimed at providing a compass to better orient within the broad horizon of new joining processes for MMHSs with an outlook for future trends.

## 1. Introduction

The use of high-performance materials such as techno-polymers and fiber-reinforced thermoplastic are opening new possibilities in terms of the “Circular Economy” concepts. Fiber-reinforced thermoplastics are distinguished by their intrinsic recyclability and re-processability. Indeed, the ability to reshape the part using thermoforming may result in a substantial reduction in material waste. Thermoplastic composites are increasingly being used to replace thermosetting composites in many commercial applications because of their advantages mentioned above. Besides, the automobile and aviation industries are sought to replace metal components with thermoplastic composites to minimize vehicle weight. As a result, thermoplastic composites are used as composite stiffeners to strengthen components, resulting in a greater strength-to-weight ratio, lighter and tougher structures, and improved fatigue properties. Thermoplastic composite stiffeners are more resistant to corrosion as compared to thermoset ones. Besides, they are produced through Out-of-Autoclave (OoA) processing, which saves cycle times and energy greatly.

Thermoplastic composites and high-performance thermoplastics are usually joined to metallic parts in complex assemblies (such as a vehicle body). Thus, hybrid metal-composite and metal-plastic systems are involved in many applications. The chemical composition, mechanical properties, and physical features of the dissimilar materials often make combining metals-polymer and metal-composite relatively difficult. The commonly used joining technologies, typically, adhesive bonding and mechanical fastening, involve a lot of shortcomings and drawbacks. For example, pretreatments are needed for adhesive bonding to remove surface oil, grease, powder, and surface oxide. This requires the use of inefficient and environmentally destructive processes. Adhesive bonding also requires advanced instruments such as fixtures and jigs (specially built and manufactured) to keep the components together during the healing process. Furthermore, adhesive bonding is susceptible to long-term instability as well as serious environmental vulnerability and shows high sensitivity to the loading direction [1,2]. Besides, both mechanical fastening and adhesive bonds increase the overall weight. The production of BMW i3, for example, uses 16 kg of adhesive in its carbon-fiber-reinforced plastic (CFRP) frame and plastic body panels, which partly reduces the weight-saving ability of composites.

Mechanical fastening procedures usually require spot contacts, which result in stress concentration and fiber interruption in composites. The adoption of inserted third parts (such as bolts or rivets) in mechanical fastening cause overweighting and increased costs. Besides, bolt-nut connections may involve problems concerning loosening fastening force owing to uneven stress distribution and concentration at threads [3,4]. Finally, mechanical fastening requires hole drilling that requires further machining and extends the overall processing time. Even though drilling operations can be substituted by hole punching processes [5], which are characterized by similar mechanical behavior and much shorter processing time, mechanical joints still require several operations including precise positioning of the components, hole drilling/punching, insertion of the connecting element, and forming of the undercuts which fasten the components.

Recently, alternative solutions have been proposed to overcome the above-mentioned limitations. Advanced mechanical joining processes represent a suitable solution. These processes, which include mechanical clinching and self-pierce riveting, do not require pre-drilled holes; thus, they enable a sensible reduction of the overall joining time.

Thermomechanical interlocking is another viable solution for hybrid metal-composite structures. These processes include friction self-riveting [6], friction riveting [6,7], fused deposition modeling-based joining [8], friction stir lap welding [9,10], injection clinching joining [11], friction stir interlocking [12,13], and friction-based filling stacking [14]. This kind of process typically needs a high degree of thermomechanical deformation of the material to be joined to achieve mechanical interlocking at a macroscopic scale.

The third category of joining processes for hybrid metal-composite structures is thermomechanical joining processes (TMJPs) enabled by micromechanical interlocking, adhesion, or chemical bonds at the joint interface. The formation of the joints is often supported by more than one joining mechanism. Some examples of TMJPs are direct laser joining [15,16,17,18,19,20], ultrasonic welding [21,22,23], friction spot joining [24,25,26,27,28], friction lap welding [29,30], friction assisted joining [31,32,33,34,35], hot press joining [36], resistance spot joining [37] and injection molding [38,39]. All these TMJPs are achieved through the application of heating and compression pressure at the joint interface. The major difference among these TMJPs is how the heating and compression pressure is generated.

The growing adoption of hybrid structures would benefit from a systematic view of the newly developed manufacturing processes. This could enable an easier and more appropriate selection of the processes for a given demand. In recent years, many literature reviews have been presented with the aim of “put in order” the available processes. In 2009, Amancio-Filho and dos Santos [40] performed a state-of-the-art study concerning the joining processes available for polymer-metal hybrid structures. Huang et al. [41] performed a literature review focused on friction stir welding of polymers, composites, and hybrid structures. Eshtayeh et al. [42] conducted a state-of-the-art study concerning the clinching of dissimilar materials. More recently, Lambiase et al. [43] have compared different friction-based processes for joining hybrid structures. These review articles have greatly contributed to the understanding of current trends and possible improvements of hybrid structure manufacturing. However, these earlier review articles are either not up-to-date or focus narrowly on some specific class of joining processes.

The main purpose of this study is to provide a comprehensive overview of available joining processes for metal-composite and metal-polymer hybrid structures. This would lead to a better selection of the suitable joining process to meet the given application demand. Thus, a critical overview of advanced joining processes for metal-composite hybrid structures developed recently on the aspect of principles, applications, advantages, and limitations of the joining processes is performed.

**Figure 1:**
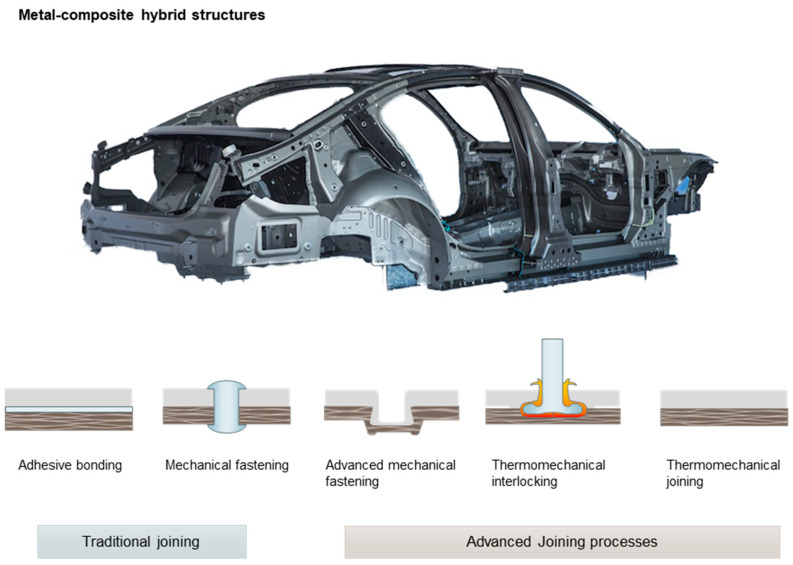
Major joining processes for hybrid metal-composite structures.

This review proposes a new classification of the joining solutions based on the main similarities among the processes. This classification includes traditional joining processes (adhesive bonding and mechanical fastening) and advanced joining processes, which include advanced mechanical fastening, thermomechanical interlocking, and thermomechanical joining, as depicted in Figure 1. The traditional joining process is out of the scope of this article and will not be introduced in detail. On the other hand, advanced joining processes are treated in different sections of the review.

## 2. Advanced Mechanical Fastening Processes

These processes involve macro-mechanical interlock between the two (or even three) components. The interlock is achieved through the plastic deformation of the joining partners or the local fracture of the composite without the need of pre-drilled holes. This group mainly includes mechanical clinching and self-pierce riveting. Additionally, in the case of clinching, an external fastening element is needed.

### 2.1. Mechanical Clinching

**Figure 2:**
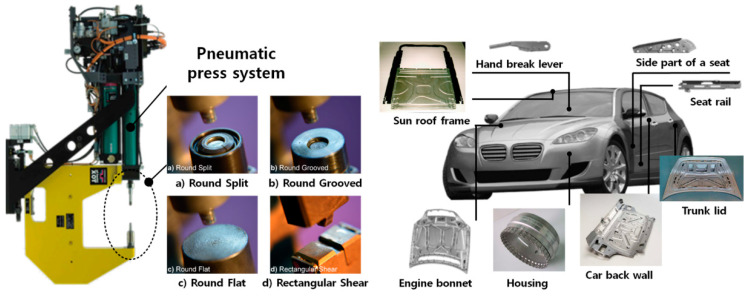
Mechanical clinching facility and its applications in automotive fabrication.

Mechanical clinching is a joining method in which two or more sheet materials are locally deformed to create a geometrical interlocking by a punch and a die. The advantage of mechanical clinching is that it can be achieved by only deforming the material to be jointed without using any external element such as rivet, screw, and adhesive. Due to these characteristics, mechanical clinching has been used to join ductile metals and polymers with various thicknesses [44,45,46,47]. It also has been used to join dissimilar materials [42,48,49,50,51]. Mechanical clinching is known as an economical joining method for sheet parts due to its low cost in investment and operation [52]. The mechanical clinching facility consists of a simple pneumatic press system and insertable punch and die set [53,54], as shown in Figure 2.

**Figure 3:**
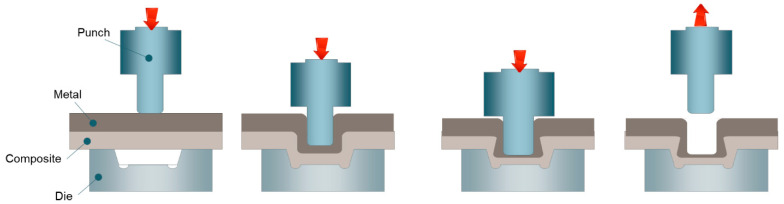
Schematic of the mechanical clinching joining process.

Figure 3 schematically shows the process of mechanical clinching [55]. The mechanical clinching can be divided into four steps: material positioning, drawing, upsetting and geometrical interlocking. Abe et al. [56] evaluated the feasibility of joining high-strength steels and aluminum alloys using mechanical clinching. They found that the applicability of mechanical clinching was mainly determined by the mechanical properties of materials, such as ductility and strength. Therefore, the tool geometry optimization for controlling the material flow at a high strain state to form geometrical interlocking is the main issue in mechanical clinching.

Many studies [57,58,59] have investigated the tool geometrical parameters that influence the quality of mechanical clinching. There are many geometrical parameters, such as punch corner, die diameter, and depth, the punch-die clearance affecting the mechanical behavior of clinched connections. The die-groove shape has been commonly reported as a critical geometrical parameter to enhance the geometrical interlocking length of the mechanical clinched joint. However, the investigation about predicting fracture of materials in actual mechanical clinching is still lacking. Lee et al. [60] have investigated the effect of tool geometry on the ductile damage value of mechanical clinched joint and Lambiase et al. [61] have predicted the fracture of materials in mechanical clinching using finite element method with damage criterion.

**Figure 4:**
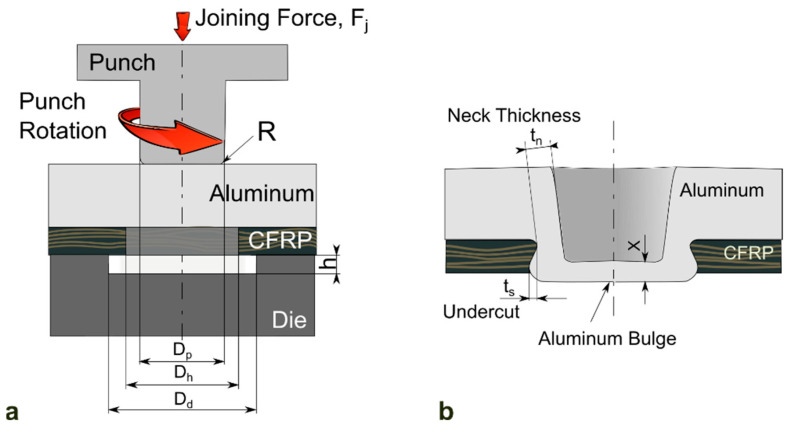
Schematic of (**a**) friction assisted (hole) clinching and (**b**) resulting geometrical interlocking of a clinched joint (Reprinted with permission from [48]).

In mechanical clinching process design, the failure mode analysis of the joint is important because it indicates the weaker part of the joint. Figure 4 shows the geometrical interlocking shape of the mechanical clinched joint. The joint strength and failure modes of mechanical clinching are determined by geometrical interlocking parameters, such as neck-thickness (t_n_) and interlocking length (t_s_), as shown in Figure 4. In general, the failure mode of the mechanical clinched joint is classified into two categories: neck fracture mode and button separation modes [62]. Neck fracture mode is caused by the large thinning of the upper material and reduction of its ductility by damage accumulation during mechanical clinching. Button separation means that the upper and lower materials are separated due to insufficient interlocking length. Lee et al. [63] developed an analytical formulation to predict the failure mode and joint strength of the mechanical clinched joint. Additionally, based on their analytical model, the required neck thickness and the interlocking length were calculated to satisfy the target joint strength. Coppieters et al. [64] proposed the analytical method to predict the pull-out strength of mechanical clinched joint by slab equilibrium techniques. This study found that the accuracy of the analytical model highly depended on the stress state of upper materials after mechanical clinching. Zhao et al. [65] and Song et al. [66] showed the finite element method with damage criterion was available to evaluate the failure behavior of mechanical clinched joints.

**Figure 5:**
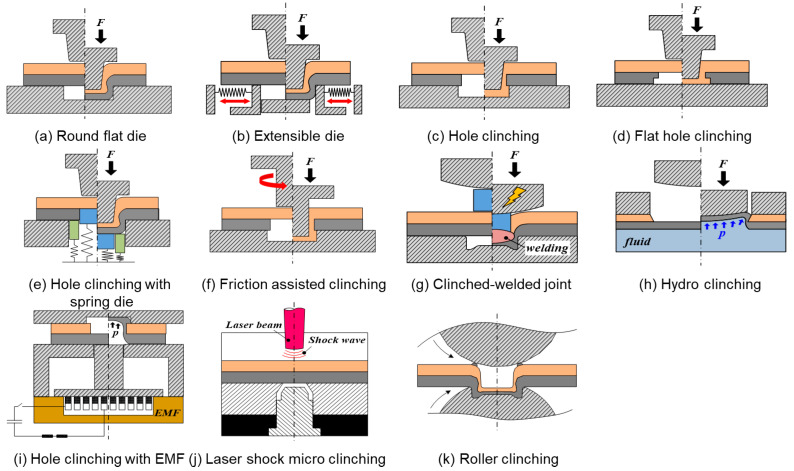
Various versions of mechanical clinching processes.

Mechanical clinching is mainly used in the civil construction and automotive industries [67] as various materials, such as advanced high-strength steels, aluminum alloys, and polymer composites, are widely used in these industries. Figure 5 shows that different kinds of mechanical clinching technologies have been developed for different materials in their combinations and applications [68]. The new trend of mechanical clinching technology development is to modify the toolsets to improve the formability of the materials. Lambiase [69] upgraded the mechanical clinching by using an extensible die to increase the formability of the materials to be joined.

Neugebauer et al. [70] introduced dieless clinching with a material heating technique for increasing the formability of magnesium alloy. Lambiase et al. [48] employed a rotation tool to heat the materials to be joined by friction energy. Another trend in mechanical clinching development is to add additional processes to address the challenges in deforming some low-ductile materials. Busse et al. [71] and Lee et al. [72] introduced mechanical clinching with sheet pre-punched on die-side to join a ductile material and a brittle material, such as advanced high-strength steel and CFRP for automotive applications. Wen et al. [73] added a re-pressing step in mechanical clinching to decrease the clinched bulge and improve the geometrical interlocking. Shi et al. [74] employed a clinching rivet process after mechanical clinching to repair the damaged mechanical clinched joint.

### 2.2. Self-Pierce Riveting

**Figure 6:**
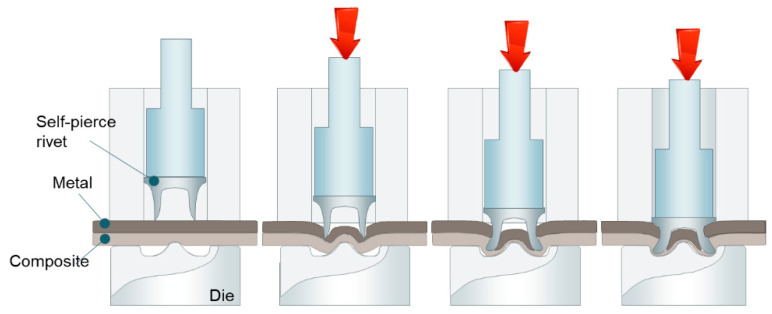
Schematic of the SPR joining process.

Self-piercing riveting (SPR) is a single-step joining technique, using a semi-tubular rivet to fasten the sheet materials. The SPR process is generally carried out in four steps [75]: clamping, piercing, flaring, and releasing, as shown in Figure 6.

**Figure 7:**
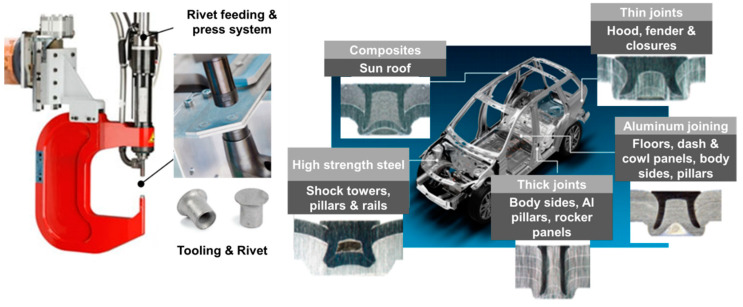
Self-piercing riveting facility and its applications in the automotive industry.

As the name suggests, a semi-tubular rivet is pressed into the sheets clamped together between a holder and die and pierces through the upper sheets. At the flaring step, the lower sheet flows into the die cavity, and the rivet flares outward to form a mechanical interlock between the upper and lower sheet. As SPR eliminates the requirement for pre-drilled or punched holes and the associate accurate alignment before joining, it allows the joints to be fabricated rapidly in a single operation. As the process relies on a mechanical interlock rather than fusion, it can be used to combine dissimilar materials without overheating. It is especially suitable for dissimilar material combinations that involve zinc-coated, organic-coated, or pre-painted steels, aluminum alloys, polymers, and composites. The unique advantages of SPR have led to a significant increase in practical applications in the automotive industry. Figure 7 shows a typical SPR machine and some of the SPR applications in the automotive industry [76,77].

**Figure 8:**
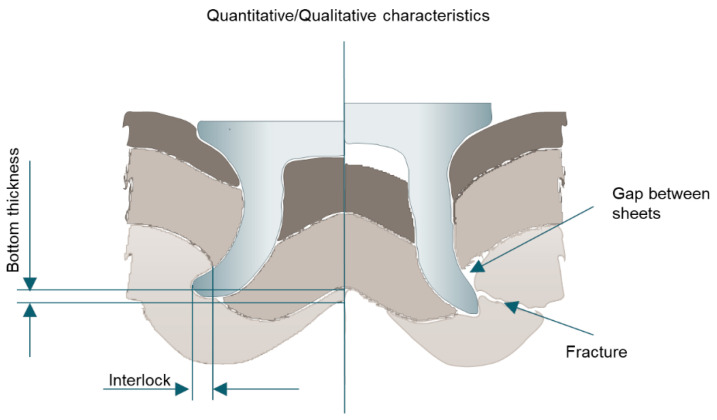
Joint quality issues in self-piercing riveting.

The joint quality of SPR is typically determined by the rivet head height, the interlock distance, and the minimum remaining bottom materials’ thickness, as shown in Figure 8 [78,79]. The rivet head height is important for the surface quality of the upper sheet. The interlock distance is the most important joint quality because it determines the strength of an SPR joint. Although the minimum remaining bottom materials’ thickness does not influence the joint strength significantly, it is important for NVH (noise, vibration, and harshness) performance and corrosion. Additionally, other joint quality aspects are considered by the defects of SPR joints, such as the rivet buckling, cracks at the lower sheet, and insufficient interlock distance.

Many process parameters can influence the quality and strength of SPR joints, such as rivet, die, and setting force. Nowadays, the rivet with a countersunk head is normally used in the SPR process. The geometry of rivet is designed by considering various factors such as the materials to be joined, die shape, and rivet hardness [80,81]. Depending on the mechanical properties of the materials to be joined, the hardness of the rivet can be in the range of 250 Hv to 600 Hv. A harder rivet should be used for joining high-strength materials. The geometry of the SPR die can affect the rivet setting force and the flaring of the rivet tail. Typically, an SPR die has a cavity with a flat bottom or a tip in the middle of the die bottom. The diameters of the SPR die cavity should be larger than that of the rivet stem to provide enough space for the rivet tail to flare. The tip in the middle of the die bottom can promote the deformation of the rivet and increases the interlock size. As SPR induces a high degree of deformations in the bottom material, a die with a tip can generate cracks when less ductile materials are used as the bottom material. SPR often requires a setting force in the range of 20–100 kN to generate sufficient mechanical interlocking between the materials to be joined.

Hou et al. [82] showed that the required setting force could be influenced by the geometry of the die and rivet. With the increased adoption of low-ductile and high-strength materials, the SPR machine will need to be built with stiffer frames and stronger servo actuators to be able to offer higher setting force during manufacturing.

## 3. Thermomechanical Interlocking Processes

Thermomechanical interlocking processes involve a high degree of thermo-mechanical deformation to generate hooks, interlocks, and to undercut the mechanically interlocking/fastening of the two components. The adoption of a heating source during the process reduces the forming forces and improves the formability of the materials involved. Currently, thermomechanical interlocking processes include friction riveting, friction self-riveting, injection clinching joining, friction-based filling stacking, friction stir lap welding, and fused deposition modeling-based joining.

### 3.1. Friction Riveting

**Figure 9:**
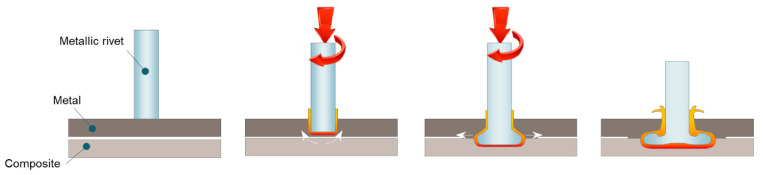
Schematic of the friction riveting process.

In friction riveting [83], a rotating metallic rivet is plunged through the metal components sitting on the top of a polymer-based component. No pre-drilled hole is necessary for the process. Once the rotating rivet is inserted into the polymer-based component whose thermal conductivity is low, the local temperature increases at the rivet tip and causes the rivet tip to expand locally, forming an anchor inside the polymer-based component, as depicted in Figure 9.

Friction riveting is suitable to connect polymer or polymer composites to different metals for a variety of applications in aerospace, automobile, and civil engineering [84]. The process was invented and patented at the Helmholtz-Zentrum Geesthacht [85]. The friction rivet process can be achieved by either a specially designed machine [86] or other well-developed equipment such as friction welding machines, milling machines, or drilling machines [87,88].

### 3.2. Friction Self-Riveting

**Figure 10:**
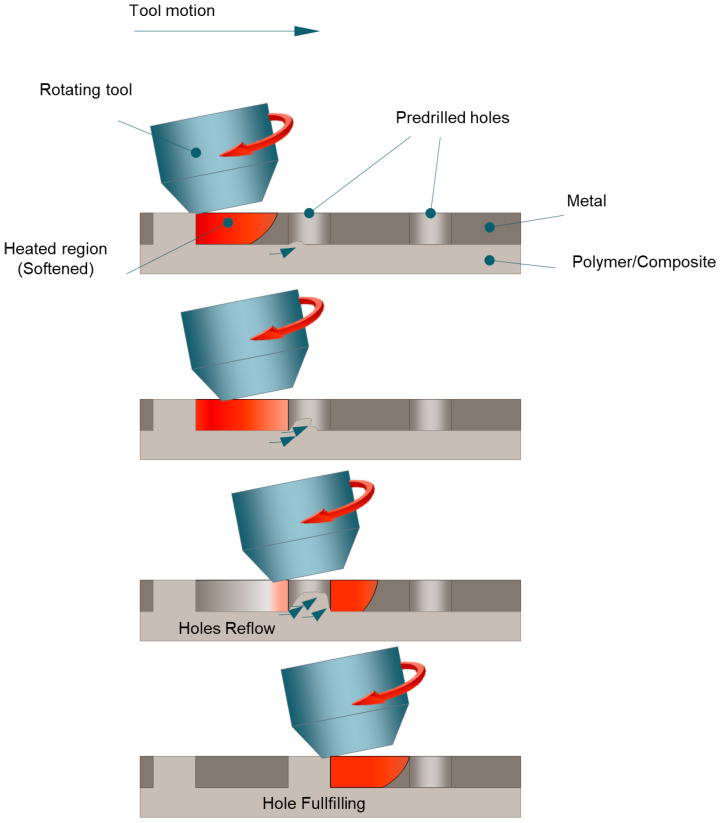
Schematic of the hole fulfilling development during friction self-riveting.

Friction self-riveting is another thermomechanical interlocking process that exploits the micro or macro holes on a metal sheet [6]. This process represents a variant of friction lap welding which needs pre-drilled holes [30,89,90,91]. Similar to friction lap welding, the process is aimed at producing continuous lap joints and involves a rotating tool that presses the surface of the top metal sheet. The interaction of the tool with the metal sheet results in frictional heat and a plunging force. The polymer (or the polymeric matrix in the composite) softens and flows towards the pre-drilled holes produced in the metal sheet. The bonding of the components is thus achieved by sufficient filling of the pre-drilled holes leading to a combination of adhesive bonding, and macro interlocking. Friction self-riveting requires that the metal sheet is pretreated through drilling to produce macro holes. A schematic of the process is depicted in Figure 10.

### 3.3. Injection Clinching Joining

**Figure 11:**
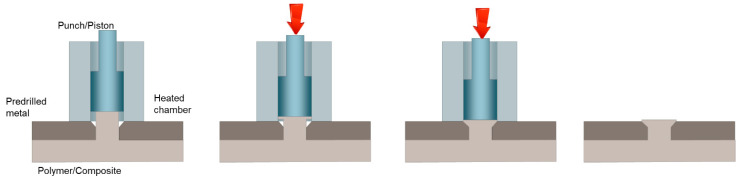
Schematic of the injection clinching joining process.

Injection clinching joining was used to produce spot joints between a metal with a pre-drilled hole and a polymer-based component [11]. The polymer-based component contains a pre-assembled protruding stud that fits into the pre-drilled hole on the metal part. A heated chamber (or eventually a rotating tool) is adopted to heat up and soften the stud, which is then compressed by a punch/piston within the heating chamber. The geometry of the chamfered pre-drilled hole as well as the height of the stud needs to be optimized to produce the needed undercut that fastens the two components. Figure 11 schematically depicts the process and procedures of an injection clinching for producing a metal-polymer hybrid structure.

### 3.4. Friction-Based Filling Stacking

**Figure 12:**
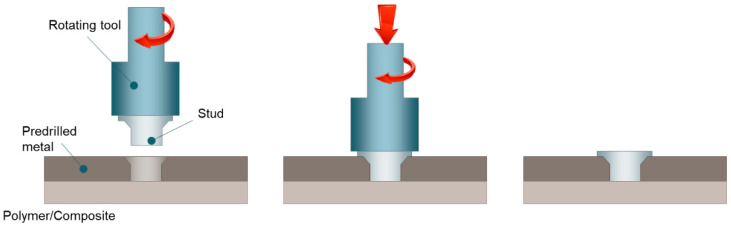
Schematic of the friction-based filling stacking process.

Friction-based filling stacking [14] was developed based on the concept of injection clinching joining but eliminates the need for the pre-assembled protruding stud (Figure 12). In friction-based filling stacking, the undercut is produced by joining an external stud that is held on a rotating tool to the polymer-based joining partner through a pre-drilled hole on the metal joining partner. The rotating tool inserts the stud through the pre-drilled hole on the metal component and plunges the stud against the underlying polymer-based component. The stud adheres to the polymer-based component and fastens the components together almost likely as a rivet.

A variant of the process that involves the direct deposition of polymeric material through fused deposition modeling (FDM) has been recently developed to avoid the use of a pre-prepared stud [8]. In fused deposition modeling, the through-hole produced on the metal joining partner is filled by the molten material exiting from an extrusion head of an FDM machine. The advantages of the process lie in the absence of forging force, and great scalability (as the stud is progressively deposited and the possibility to use different stud geometries beyond circular). However, only preliminary results on low-performing filaments (polypropylene) were reported and the produced joints were characterized by relatively lower joint strength (5–6 MPa).

### 3.5. Friction Stir Lap Welding

**Figure 13:**
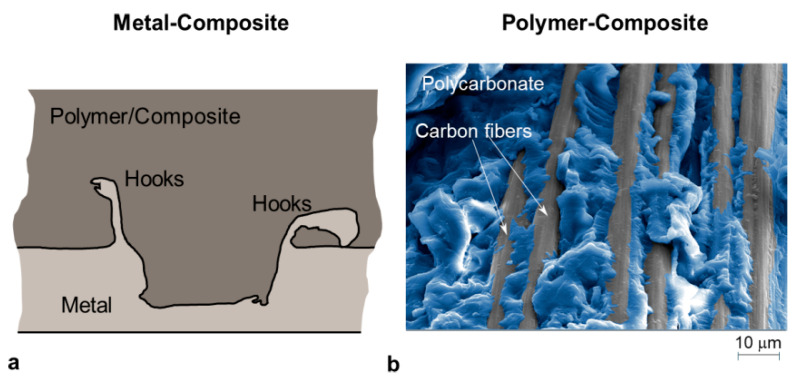
(**a**) Schematic of the cross-section of a metal-composite joint made by FSW and (**b**) fracture surface highlighting the adhesion of polycarbonate to the carbon fibers of a composite laminate (CFRP with epoxy matrix).

Friction stir lap welding, which was originally developed for welding hard-to-weld metals (such as aluminum alloys), has been extended to various combinations of materials (e.g., metal-composite, metal-polymer as well as polymer-composite). Different joining mechanisms may be involved such as the adhesion of a polymer to the fibers of the second, as shown in Figure 13b (composite material) [92,93]. In the case of metal-composite joints, the metal is generally positioned below the composite laminate. The rotating probe stirs against the metal surface leading to the formation of hooks that fastens the two components [9,10,41], as depicted in Figure 13a.

## 4. Thermomechanical Joining Processes

**Figure 14:**
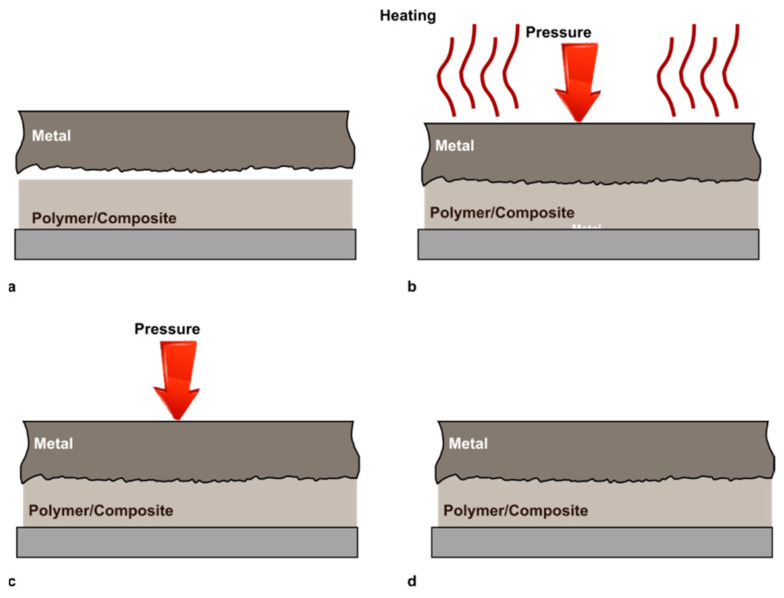
Schematic of a thermomechanical joining process: (**a**) Positioning; (**b**) Heating and pressing; (**c**) Consolidation; and (**d**) Finished joint.

Thermomechanical joining processes (TMJPs) do not require substantial deformation of the components. They use an external energy source (such as a laser, friction, or ultrasonic displacement) to heat the joint interface until it reaches the desired joining temperature range. An application of compression is often desirable to achieve higher bonding strength. The sequence of a TMJP is schematically illustrated in Figure 14.

The joint strength can be affected by many factors including but not limited to:

- The surface conditions of the joining partners including the nature of the polymer (polymer matrix), the chemical composition of the metal surface, and the morphology of the metal surface.
- The joining parameters used during the operation.

Depending on the equipment adopted, these processes can be used to produce spot and continuous connections.

### 4.1. The Surfaces of the Joining Partners

Micromechanical interlocking, physical interactions and chemical bonds may be used to connect metal to a composite component. The formation of the joints is often supported by more than one mechanism. Micromechanical interlocking can be achieved by localized deformation of the metal that flows around the carbon fibers [21,94] or the flow of the molten polymer into natural or artificial asperities on the metal surface [29,95]. Roughing the metal surface can be used to enhance the mechanical interlocking. It has been shown that suitable surface textures (groves, pores, or protrusions) on the metal faying surface can significantly enhance the bonding between the metal and the polymer-based materials through the formation of micromechanical interlocking at the joint interface.

The atomic-level interaction between metal and polymer at the joint interface can be classified into two categories: physical interactions and chemical bonding. Physical interactions include the generation of Van der Waals force and hydrogen bonding. Van der Waals force is the weakest force among the atoms in direct contact at the joint interface and it is the results of London interaction, Keesom interaction, or Debye interaction. Hydrogen bonds can be produced at the joint interface when hydrogen atoms on the polymer faying surface interact with the metal surface. Compared to chemical bonds, hydrogen bonds are also much weaker. Chemical bonding at the polymer-metal interface involves the formation of covalent bonds. Table 1 summarizes the various forms of bonds, their bonding force, and equilibrium duration at room temperature to give a clearer understanding of the difference between the bonds.

The atomic/electronic level interaction between metal and polymer during joining has not been fully understood. This is because the interaction occurred at elevated temperatures at the buried joint interface. It is hard to in situ detect the buried chemical information during dissimilar material joining, and it is also extremely difficult to analyze the interfacial chemical information of the as-joined hybrid structures at the atomic or electronic level.

Recent investigations [29,96] showed that considerable bonding strength is achievable when a polymer is welded to a metal with a relatively smooth surface. To reveal the underpinning bonding mechanism, Liu et al. [29] adopted X-ray photoelectron spectroscopy (XPS) to determine the binding states of the elements at the metal-polymer interface. The results showed that the carbonyl components (C=O) on the polymer surface were the primary reaction sites for developing chemical bonds at the polymer-metal interface. This is evidenced by the decrease of C=O components and the formation of new C–O–Al components at the metal-polymer interface [29].

The importance of carbonyl groups for promoting metal-polymer bonding was further highlighted through a systematic analysis of available literature. The results were summarized in Table 2. The thermoplastics which contain carboxyl groups (including PA, PET, PEI, PC, and PMMA) could be directly welded to different metals without special surface modification. In contrast, the thermoplastics which do not contain carboxyl groups (including PE, PP, ABS, PVC, and PPS) need a special surface pretreatment to generate strong bonding between the polymers and the metal. The polymer can be modified by adding oxygen atoms into the polymer for generating C–O–M chemical bonds during welding.

Various surface modification methods are under development in this field to enhance the bonding between metal and polymer. The surface modification of the polymer side is to add oxygen atoms or ions on polymer chains to promote the generation of chemical bonds between metal and polymer during TMJP. In contrast, the objective of pretreatment on a metal surface is often to generate micro holes or textures for generating micromechanical interlocking between the joining partners at the joint interface.

### 4.2. The Processing Conditions

Although similarity exists between TMJP and adhesive bonding, these two processes are different in principle. TMJPs are developed to generate a bonding at the interface between a thermoplastic polymer and a metal under the activation of heating and compression pressure. TMJPs aim to form strong chemical bonds between the polymer matrix and metal in addition to developing micromechanical interlocking and physical interaction at the joint interface. In contrast, adhesive bonding between a metal and a polymer component is achieved by adding a viscous liquid polymer at the interface and developing the bonding through the evaporation of a solvent or through curing via heat, time, or pressure. Therefore, the adhesive bonding is formed between a metal and a thermoset. Compared to adhesive bonding, TMJPs has the following advantages:

- TMJPs can complete rapidly while adhesive bonding needs a long curing time.
- No autoclave is necessary.
- No additional thermoset interlayer. This may solve numerous issues, including weight increase, cost, and storage.
- Thermoplastics are commonly tougher as compared to thermosets.
- Damaged thermoplastic joints could be restored by applying TMJP without the need to disassembling the structure.

Although promising, TMJP needs to overcome many challenges to enable its high-volume engineering applications. Since the thermoplastic material needs to be heated to flow to achieve the bonding during TMJP, sufficient heat is necessary to molten the polymer at the joint interface. An overheating at the joint interface will cause a significant polymer degradation and therefore it needs to be avoided. Therefore, correct TMJP parameters are critical for producing high-quality dissimilar material joints. This dramatically complicates the TMJP process design and requires a deep understanding of the materials to be joined and their response at elevated temperatures.

**Figure 15:**
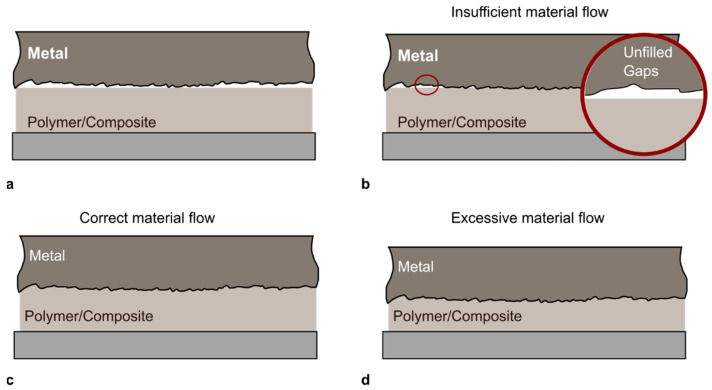
Schematic of the adherends surfaces: (**a**) Before joining; (**b**) Insufficient; (**c**) Correct and (**d**) Excessive material flow.

These concerns are evidenced by numerous experimental studies which showed that the processing temperature played a key role in affecting the strength of the joints made by TMJPs. Joining at a temperature lower than the target temperature results in inadequate energy to activate the joining. Overheating, on the other hand, can cause negative effects such as formation of voids, higher thermal shrinkage, and significant polymer thermal degradation. This may have a major effect on the joints’ mechanical and chemical properties. Besides, joining at higher temperatures may lead to energy waste and prolonged processing time. The flowability of the polymer to be jointed is mainly determined by the temperature at the joint interface. The temperature should be adequate to melt the polymer for the complete filling of any spaces between the metal and polymer (see Figure 15b,c). However, as indicated in Figure 15d, an excessive material flow may have a substantial effect on the geometrical resistance of the components being coupled (e.g., excessive thinning, material reflow, and the coplanarity between the adherends’ surfaces).

**Figure 16:**
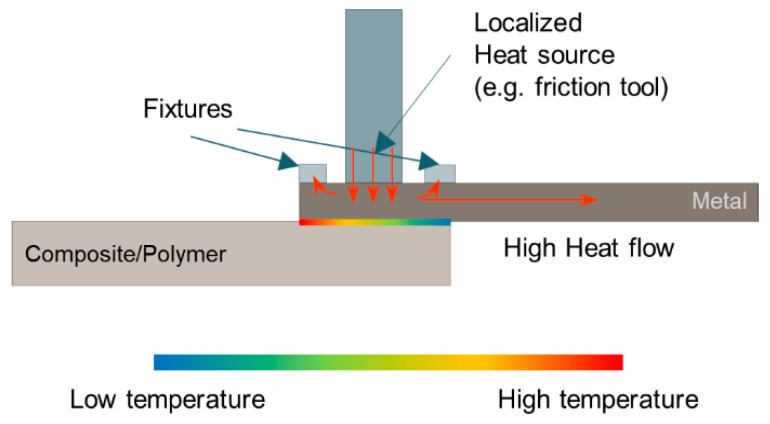
Schematic of uneven temperature distribution produced at the metal-composite interface since the localized heating source, high heat flow in metal, and heat exchanges with fixtures.

During the TMJP, the temperature distribution at the metal-composite interface is not uniform. Some setup of the joining processes may lead to uneven temperature distribution, as depicted in Figure 16, including:

- a limited dimension of the heating source on the metal component surface;
- the presence of clamping equipment that promotes localized heat exchanges and
- the limited contact interface between the metal and the underlying polymer/composite.

This leads to uneven joining conditions at the metal-composite interface [31,121]. To better understand the influence of the process parameters as well as to improve the process design, recent studies investigated the temperature distribution and history during thermomechanical joining processes [18,34] to determine the optimal processing conditions for a given material pair.

However, since numerous process parameters are involved, the optimization of these processes based on a full experimental approach would be costly and time-consuming. Additionally, this would lead to local maxima rather than the effective optimization of the process. Thus, a viable solution for process design is represented by the development of models that, once validated over a wide processing space, would provide accurate predictions of the processing conditions (e.g., temperature at the metal-composite interface). The process design and optimization could be performed by validated simulation campaigns using these validated numerical [35] and Machine Learning [122] models.

## 5. Discussion

With the wide adoption of hybrid metal-composite structures, developing advanced joining methods suitable for hybrid structures has become a major challenge. Traditional joining methods, such as mechanical fastening and adhesive bonding, exhibit significant drawbacks. Fast mechanical joining, thermomechanical interlocking, and thermomechanical joining techniques have shown their capability to produce high-performance joints with reduced manufacturing time. The advantages provided by these processes could potentially make it possible to overcome almost all the limitations involved in traditional mechanical fastening and adhesive bonding techniques. These processes provide advantages of higher efficiency, easier standardization, easier automation, short joining time, and less environmental effect than adhesive bonding. Furthermore, the TMJPs do not need to destroy the external surface of the polymer-based joining partner, resulting in a relatively smooth surface. This feature is attractive for many engineering applications compared to mechanical fastening methods, which typically need to produce a through-hole on the parts to be joined. A common benefit of most of the new joining processes mentioned above is that they do not need an additional connecting part, which helps to reduce weight. A comparison among the three classes of advanced joining processes is summarized in Table 3. Here, some reference values of the ultimate shear force are also reported. However, this is only indicative as different materials in pairs were used in the research. 

Besides, many of the presented processes have been developed only recently, and consequently, they are still far from being optimized. This makes it harder for a direct comparison among the processes. When dealing with metal-composite laminates’ joining, advanced mechanical fastening processes may cause severe composite damage (e.g., delamination). This is more often observed in clinched connections; however, hole-clinching, and self-pierce riveting were less affected by this issue. The metal formability may also represent an issue when using mechanical fastening processes. However, recent studies showed that heat-assisted systems (e.g., friction assisted clinching) [48], are helpful for eliminating the process-induced defects (fracture at the metal neck). To better understand the damage in the composite laminate, numerical models involving failure criteria of the composite are highly demanded. This would enable to explore more solutions (e.g., geometrical features of the tool), with a greater understanding of the influence of the process parameters on the joint’s quality (morphology, damage, mechanical strength).

Advanced joining processes share a common demand: the necessity to develop reliable models for the prediction of the process-induced defects as well as the mechanical behavior of the joints. So far, the process design has been mainly based on extensive and time-consuming experimental campaigns that generally lead to local optima and do not provide significant transferable knowledge, as the strength of the joints are related to the processing parameters using “black-box” models. On the other hand, the development of numerical models of the processes would enable a better understanding of the phenomena involved. At the same time, it could represent a precious tool for determining the influence of process parameters on the quality of the connections.

For advanced mechanical fastening processes, the process-induced deformation determines the dimension of the undercut and the thickness of the necks. These have been extensively studied through numerical simulations when dealing with metal joining [61]. However, there is a substantial lack of knowledge about the influence of process parameters on process-induced defects when composite laminates are involved. This would represent the key element for proper process development in the next few years. Thermomechanical interlocking processes also require the development of a suitable numerical model that is capable of predicting the material flow as well as the thermal evolution during the process.

These models would enable the prediction of the dimension of macro-interlocks. Besides, the prediction of the temperature field could predict the onset of temperature-induced defects, which include a variation of crystallinity, development of porosities, and even thermal degradation of the polymer. However, the development of suitable and reliable numerical models is even more complex since the high amount of deformation, which is typically involved in these processes. Besides, it would also require a comprehensive characterization of the polymer (or composite) to determine the temperature-dependent mechanical behavior. This would enable to determine the material flow during the joining process more accurately.

Thermomechanical joining processes would also benefit from the adoption of numerical models for a better understanding of the influence of the process conditions on the quality of the joints [121]. The quality of these joints is mainly temperature-dependent. The joining mechanism as well as the main defects are mainly determined by the temperature reached at the interface [128]. Besides, these processes involve relatively low material flow as compared to advanced mechanical fastening and thermomechanical interlocking processes. This has greatly simplified the development of suitable models and the identification of optimal processing conditions. Several studies were aimed at developing and validating numerical models for thermomechanical joining processes. The development of such models is simpler when dealing with laser-assisted joining [18,34] where the power is an “explicit” process input; while it is more difficult when dealing with friction-based processes as reported in [35,122]. Although the promising results were provided by these studies, the process simulation meets great challenges when dealing with metal-composite laminates. Indeed, the presence of the fibers that locally hinder the polymer flow as well as the metal interaction with the fibers, which may lead to metal deformation and local mechanical interlocking, would require more sophisticated models.

Even though a direct comparison is hard to be performed, the main pros and cons of each process are summarized in Table 4. Advanced mechanical fastening processes generally produce medium-strength spot joints. Particularly, self-pierce riveting demands an external connection element, which increases the cost and weight. On the other hand, clinching may be affected by the formability of the involved metal sheet [48]. These processes are extremely fast (typical joining time of 0.5 s) and easy to be performed and do not involve temperature increase and related issues. In particular, despite the increasing use of high-strength materials to reduce the weight of structures, it is required to improve the joining efficiency (bonding strength versus base material strength) for advanced mechanical fastening to satisfy the safety and durability at the joining parts of hybrid structures. The increase in cost due to additionally using joining elements to improve joinability for hybrid material structures will also be a factor that determines the popularization of advanced mechanical fastening soon.

Thermomechanical interlocking behaves like an alternative to conventional riveting processes (except friction stir welding). The formation of thermomechanical interlocking is based on the mechanical interlock between the metal and the composite employing a stud/rivet. This is formed or joined by employing a thermomechanical process that enables to fill the cavities pre-drilled on the metallic sheet through an external stud (as in friction-based filling stacking), a deposited material, or the thermomechanical upsetting of the protrusion (as for injection clinching joining). All these processes, (except for friction stir welding) produce spot connections that are prone to introduce high-stress concentration.

Thermomechanical joining processes are based on the bonding of the thermoplastic matrix (or a thin layer of thermoplastic, as proposed in [129,130,131]) and the metal sheet. The bonding process involves heating the polymer above the softening/melting point to achieve intimate contact and bonding through different mechanisms (Van Der Waals forces, chemical bonding, and micro interlocking). These processes can be performed through different heating sources including laser, friction, ultrasonic, etc., and can be exploited to produce spot joints or continuous joints.

## 6. Conclusions

Hybrid metal-polymer and metal-composite structures represent the key solution in the automotive and aircraft industries to reduce product weight. Many efforts are being conducted to provide alternative solutions to established joining techniques. Emerging joining technologies are gaining great attention as they would provide many advantages over conventional mechanical fastening and adhesive bonding, including inherent simplicity, high joint strength, short joining time, no embedded materials, and high automation and standardization viability. However, a systematic view of this process was still missing. This review article would be beneficial for both process developments and for process selections for metal-polymer and metal-composite structures. Based on their characteristics, advanced joining processes have been classified into three main categories:

- Advanced mechanical fastening: the joining is achieved through mechanical interlocking, which is produced through plastic deformation (generally at room temperature) of the joining partners. These processes produce a significant alteration at both sides of the connection (protrusions, defects, etc.) Some processes do not require hole drilling as well as external connecting elements.
- Thermomechanical interlocking: the joining is mainly developed through macro-mechanical interlocking and high thermoplastic deformation of the joining partners. These joining processes damaged the appearance of at least one joining partner due to the high degree of thermoplastic deformation as well as the adoption of protruding elements in many cases. These joining processes can be used to produce either spot or continuous joints.
- Thermomechanical joining processes: the joining is achieved through the application of heating (different heating media are available) and compression pressure at the joint interface. Different joining mechanisms can be activated including chemical bonding, physical bonding, and micro-mechanical interlocking. These processes can guarantee better joint appearance since high degree of plastic deformation of the joining partners can be avoided during the joining. These processes can also be used to produce either continuous or spot joints.

After a description of the processes, and their peculiarities, an analysis of the main advantages, shared issues, as well as current limitations in process design and simulation were provided. Due to their complexity and relatively recent development, a shared need to almost all these processes is the development of a robust design and process simulation tools. This would greatly advance the understanding of the process, and promote process development and optimization. Finally, an outlook on current trends and future developments has been provided.

## Figures and Tables

**Table 1:** Different types of bonds and their range of bonding energy and interaction range.

Bond Type	Equilibrium Length (nm)	Bonding Energy (kJ mol^−1^)
Ionic (primary, chemical)	0.2–0.4	560–1000
Covalent (primary, chemical)	0.1–0.3	60–800
Metallic (primary, chemical)	0.2–0.6	100–350
Hydrogen (secondary, physical)	0.3–0.5	50
London (secondary, physical)	0.3–0.5	1–40
Debye (secondary, physical)	0.3–0.5	2
Keesom (secondary, physical)	0.3–0.5	2–8

**Table 2:** A summary of the joinability of metal and polymer combinations.

Authors	Polymers	Metals	Joining Methods	C=O Groups	Specific Surface Modification
Liu et al. [30]	PA	6061 Al	FLW	Yes	No
Liu et al. [91]	PA	AZ31B Mg	FLW	Yes	No
Liu et al. [90]	PE	Non-combustible Mg	FLW	No	Plasma electrolytic oxidation
Nagatsuka et al. [97]	CFRP-PA	5052Al	FLW	Yes	No
Nagatsuka et al. [98]	PA	Low carbon steel	FLW	Yes	No
Wu et al. [99]	CFRP-PA	Copper	FLW	Yes	No
Nagatsuka et al. [100]	CFRP-PA	304 stainless steel	RSW	Yes	No
Nagatsuka et al. [100]	CFRP-PPS	304 stainless steel	RSW	No	Coupling agent
Nagatsuka et al. [100]	CFRP-PP	304 stainless steel	RSW	No	Acid-modified
Ageorges et al. [101]	PEI	7075 Al	RSW	Yes	No
Katayama et al. [102]	PET	304 stainless steel	LDJ	Yes	No
Kawahito et al. [103]	PET	Zr55Al10Ni5Cu30	LDJ	Yes	No
Farazila et al. [104]	PET	copper	LDJ	Yes	No
Farazila et al. [104]	PET	5052 Al	LDJ	Yes	No
Wahba et al. [105]	PET	AZ91D Mg	LDJ	Yes	No
Jung et al. [106]	CFRP-PA	304 stainless steel	LDJ	Yes	No
Jung et al. [107]	CFRP-PA	Galvanized steel	LDJ	Yes	No
Yusof et al. [108]	PET	5052Al	LDJ	Yes	No
Hussein et al. [109,110]	PMMA	304 stainless steel	LDJ	Yes	No
Lambiase et al. [19]	PC	304 stainless steel	LDJ	Yes	No
Zhang et al. [111]	CFRP-PA	6061Al	LDJ	Yes	No
Ai et al. [112]	PET	Ti6Al4V	LDJ	Yes	No
Chan et al. [113]	PET	CP Ti	LDJ	Yes	No
Jung et al. [114]	ABS	Galvanized steel	LDJ	No	Surface oxidation
Yusof et al. [115]	PET	5052Al	FAW	Yes	No
Amancio et al. [25]	CFRP-PPS	AZ31 Mg	RFSSW	No	Acetone rinsing
Goushegir et al. [26]	CFRP-PPS	2024 Al	RFSSW	No	Acetone rinsing
Esteves et al. [116]	CFRP-PPS	6181 Al	RFSSW	No	Acetone rinsing
Balle et al. [117]	CFRP-PA	1050Al	USW	Yes	No
Balle et al. [118]	CFRP-PA	5754Al	USW	Yes	No
Lionetto et al. [23]	CFRP-PA	5754Al	USW	Yes	No
Lambiase et al. [119]	PEEK	5053Al	FAW	Yes	Laser texturing
Lambiase et al. [120]	PVC	5053Al	FAW	No	Laser texturing

Nomenclature: FLW (Friction lap welding); RSW (Resistance spot welding); LDJ (Laser direct joining); FAW (Friction assisted welding); RFSSW (Refill friction stir spot welding); USW (Ultrasonic spot welding); PA6 (Polyamide6); PE (Polyethylene); CFRP-PA6 (Carbon fiber reinforced plastic with Polyamide6 as matrix); CFRP-PPS (Carbon fiber reinforced plastic with poly phenylene sulfide as matrix); CFRP-PP (Carbon fiber reinforced plastic with polypropylene as matrix); PEI (Polyetherimide); PET (Polyethylene terephthalate); PMMA (Polymethylmethacrylate); PC (Polycarbonate); ABS (Acrylonitrile butadiene styrene); CFRP-PA66 (Carbon fiber reinforced plastic with Polyamide66 as matrix); PEEK (Polyether ether ketone); PVC (Polyvinylchloride).

**Table 3:** Main characteristics of advanced joining processes.

Main characteristics	Advanced Mechanical Fastening	Thermomechanical Interlocking	Thermomechanical Joining
Main joining mechanism	Macro-mechanical. interlocking	Macro-mechanical interlocking.	Micromechanical interlocking. Chemical bonding.
How the joints are achieved	Moderate plastic deformation.	Large thermoplastic deformation.	Application of compression pressure and heating.
Main limitations/issues	Damage of the composite (when the process is performed without pre-drilled holes). Formability of the metal may lead to fracture (this can be solved by the adoption of heating systems).	The complexity of the process. May involve temperature-induced issues. Heavy joining forces.	The complexity of the process. Temperature-induced defects. Uneven joining conditions.
Process design	Difficulty to use FE models to design the tools since the difficulty to predict the composite behavior/damage.	Since the composite is often not deformed, numerical simulations can be used for design purposes.	Thermo-mechanical simulations enable to predict accurately the processing conditions (stress, temperature).
Type of joint	Spot.	Spot/continuous.	Spot/continuous.
Static behavior-ultimate shear force [kN]	Clinching 3-kN [123]. Self-pierce riveting up to 12 kN [124].	Frict. riveting: 7 kN [83]. Frict. stir welding 3 kN [6]. Frict. based filling 1.2 kN [14]. Frict. based stacking 1 kN [125].	Frict. assisted joining: 11 kN [31]. Frict. spot joining: 2.5 kN [27]. Ultrasonic welding: 8 kN [21]. Laser-assisted joining: 10 kN [126].
Current design limitations	The demand for modeling techniques that enable the prediction of the onset of process-induced defects (e.g., damage) in the composite laminate.	Characterization of thermo-mechanical properties of the materials involved to develop a reliable numerical model of the processes.	Characterization of thermo-mechanical properties of the material involved to develop a reliable numerical model of the process. Uneven joining conditions make it even more difficult to determine optimal joining conditions.
Key development trends	New heating systems and control. Modeling and prediction of mechanical behavior.	New process control strategies [127]. Numerical modeling of the process. Modeling and prediction of mechanical behavior.	Localized characterization systems of the joints [21]. High-speed systems for process control. Structured approach for the process design for virtual process optimization (integration of numerical models with Artificial Intelligence [122]. New surface pretreatment processes to improve the adhesion of the components.

**Table 4:** Main pros and cons of the advanced joining processes for metal-composite hybrid structures.

Advanced Joining process	Pros	Cons
**Advanced mechanical fastening processes**		
Mechanical Clinching	Easy and fast Does not involve external material/components Does not involve temperature-induced issues	Spot connection Involves composite delamination The formability of the metal can involve severe limitation to the applicability
Self-pierce riveting	Easy and fast Does not involve temperature-induced issues	Spot connection Involves composite delamination Involves an external (and expensive) joining element
**Thermomechanical interlocking processes**		
Friction Riveting	Easy and fast	Spot connection Involves an external joining element May involve temperature-induced issues Heavy joining forces
Friction self-riveting	High productivity Does not involve external material/components	Spot connection May involve temperature-induced issues Heavy joining forces
Injection clinching joining	Easy Low processing forces	Spot connection Demands of a preformed stud being produced on the polymer Requires the drilling of a hole in the metal component
Friction-based filling stacking	-	Spot connection Requires the drilling of a hole in the metal component May involve temperature-induced issues Heavy joining forces
Friction stir lap welding	Continuous joint	May involve temperature-induced issues Heavy joining forces May cause fiber interruption
**Thermomechanical joining processes**		
Radiation based processes (laser, IR, etc.)	Continuous or spot joint Negligible processing forces	Involves temperature-induced issues Surface pretreatments are highly recommended Radiation absorption The connection is limited to the interface (likely adhesive bonds)
Friction-based processes	Continuous or spot joint	Involves temperature-induced issues Surface pretreatments are highly recommended Need medium compression forces The connection is limited to the interface (likely adhesive bonds); however, deep interlocking is possible if long protrusions are made on the metal surface.

## Data Availability

Data sharing not applicable.

## References

[1] Trask R.S., Hallett S.R., Helenon F.M.M., Wisnom M.R. (2012). Influence of process induced defects on the failure of composite T-joint specimens. Compos. Part A Appl. Sci. Manuf..

[2] Khosravani M.R., Anders D., Weinberg K. (2019). Influence of strain rate on fracture behavior of sandwich composite T-joints. Eur. J. Mech. A Solids.

[3] Zhou W., Zhang R., Ai S., He R., Pei Y., Fang D. (2015). Load distribution in threads of porous metal–ceramic functionally graded composite joints subjected to thermomechanical loading. Compos. Struct..

[4] Zhou W., Ai S., Chen M., Zhang R., He R., Pei Y., Fang D. (2015). Preparation and thermodynamic analysis of the porous ZrO2/(ZrO_2_ + Ni) functionally graded bolted joint. Compos. Part B Eng..

[5] Lambiase F., Durante M. (2017). Mechanical behavior of punched holes produced on thin glass fiber reinforced plastic laminates. Compos. Struct..

[6] Meng X., Huang Y., Xie Y., Li J., Guan M., Wan L., Dong Z., Cao J. (2019). Friction self-riveting welding between polymer matrix composites and metals. Compos. Part A Appl. Sci. Manuf..

[7] Blaga L., Bancilă R., dos Santos J.F., Amancio-Filho S.T. (2013). Friction Riveting of glass–fibre-reinforced polyetherimide composite and titanium grade 2 hybrid joints. Mater. Des..

[8] Ozlati A., Movahedi M., Tamizi M., Tartifzadeh Z., Alipour S. (2019). An alternative additive manufacturing-based joining method to make Metal/Polymer hybrid structures. J. Manuf. Process..

[9] Huang Y., Meng X., Xie Y., Li J., Si X., Fan Q. (2019). Improving mechanical properties of composite/metal friction stir lap welding joints via a taper-screwed pin with triple facets. J. Mater. Process. Technol..

[10] Huang Y., Meng X., Xie Y., Li J., Wan L. (2018). Joining of carbon fiber reinforced thermoplastic and metal via friction stir welding with co-controlling shape and performance. Compos. Part A Appl. Sci. Manuf..

[11] Abibe A.B., Amancio-Filho S.T., Dos Santos J.F., Hage E. (2013). Mechanical and failure behaviour of hybrid polymer–metal staked joints. Mater. Des..

[12] Wang T., Upadhyay P., Reza-E-Rabby M., Li X., Li L., Soulami A., Kappagantula K.S., Whalen S. (2020). Joining of thermoset carbon fiber reinforced polymer and AZ31 magnesium alloy sheet via friction stir interlocking. Int. J. Adv. Manuf. Technol..

[13] Wang T., Li L., Pallaka M.R., Das H., Whalen S., Soulami A., Upadhyay P., Kappagantula K.S. (2021). Mechanical and microstructural characterization of AZ31 magnesium‑carbon fiber reinforced polymer joint obtained by friction stir interlocking technique. Mater. Des..

[14] Huang Y., Meng X., Xie Y., Li J., Wan L. (2019). New technique of friction-based filling stacking joining for metal and polymer. Compos. Part B Eng..

[15] Katayama S. (2013). Handbook of Laser Welding Technologies.

[16] Lambiase F., Genna S. (2018). Experimental analysis of Laser assisted joining of Al-Mg aluminium alloy with Polyetheretherketone (PEEK). Int. J. Adhes. Adhes..

[17] Lambiase F., Genna S. (2018). Laser assisted joining of AA5053 aluminum alloy with polyvinyl chloride (PVC). Opt. Laser Technol..

[18] Lambiase F., Genna S., Kant R. (2018). Optimization of laser-assisted joining through an integrated experimental-simulation approach. Int. J. Adv. Manuf. Technol..

[19] Lambiase F., Genna S., Leone C., Paoletti A. (2017). Laser-assisted direct-joining of carbon fibre reinforced plastic with thermosetting matrix to polycarbonate sheets. Opt. Laser Technol..

[20] Schricker K., Samfaß L., Grätzel M., Ecke G., Bergmann J.P. (2020). Bonding mechanisms in laser-assisted joining of metal-polymer composites. J. Adv. Join. Process..

[21] Staab F., Liesegang M., Balle F. (2020). Local shear strength distribution of ultrasonically welded hybrid Aluminium to CFRP joints. Compos. Struct..

[22] Yeh R.-Y., Hsu R.-Q. (2016). Development of ultrasonic direct joining of thermoplastic to laser structured metal. Int. J. Adhes. Adhes..

[23] Lionetto F., Balle F., Maffezzoli A. (2017). Hybrid ultrasonic spot welding of aluminum to carbon fiber reinforced epoxy composites. J. Mater. Process. Technol..

[24] Junior W.S., Handge U.A., dos Santos J.F., Abetz V., Amancio-Filho S.T. (2014). Feasibility study of friction spot welding of dissimilar single-lap joint between poly(methyl methacrylate) and poly(methyl methacrylate)-SiO_2_ nanocomposite. Mater. Des..

[25] Goushegir S.M., dos Santos J.F., Amancio-Filho S.T. (2014). Friction Spot Joining of aluminum AA2024/carbon-fiber reinforced poly(phenylene sulfide) composite single lap joints: Microstructure and mechanical performance. Mater. Des..

[26] Amancio-Filho S.T., Bueno C., dos Dos Santos J.F., Huber N., Hage E. (2011). On the feasibility of friction spot joining in magnesium/fiber-reinforced polymer composite hybrid structures. Mater. Sci. Eng. A.

[27] Goushegir S.M., Dos Santos J.F., Amancio-Filho S.T. (2015). Influence of process parameters on mechanical performance and bonding area of AA2024/carbon-fiber-reinforced poly(phenylene sulfide) friction spot single lap joints. Mater. Des..

[28] Jiang M., Chen K., Chen B., Wang M., Hua X., Zhang L., Shan A. (2020). Friction spot joining of TC4 alloy and ultra-high molecular weight polyethylene: Focused on temperature-force relationship. Mater. Des..

[29] Liu F.C., Dong P., Pei X. (2020). A high-speed metal-to-polymer direct joining technique and underlying bonding mechanisms. J. Mater. Process. Technol..

[30] Liu F.C., Liao J., Nakata K. (2014). Joining of metal to plastic using friction lap welding. Mater. Des..

[31] Lambiase F., Paoletti A., Durante M. (2021). Mechanism of bonding of AA7075 aluminum alloy and CFRP during Friction Assisted Joining. Compos. Struct..

[32] Lambiase F., Paoletti A. (2018). Friction Assisted Joining of titanium and polyetheretherketone (PEEK) sheets. Thin-Walled Struct..

[33] Lambiase F., Paoletti A., Grossi V., Genna S. (2017). Improving energy efficiency in friction assisted joining of metals and polymers. J. Mater. Process. Technol..

[34] Lambiase F., Genna S., Kant R. (2018). A procedure for calibration and validation of FE modelling of laser-assisted metal to polymer direct joining. Opt. Laser Technol..

[35] Lambiase F., di Ilio A., Paoletti A. (2020). Hybrid numerical modeling of Friction Assisted Joining. J. Manuf. Process..

[36] Tolephih M.H., Abood A.N., Saad H.M. (2020). Hot Press Bonding of Aluminum Alloy AA6061-T6 to Polyamide and Polyamide Composites. IOP Conf. Ser. Mater. Sci. Eng..

[37] Szallies K., Bielenin M., Schricker K., Bergmann J.P., Neudel C. (2019). Single-side resistance spot joining of polymer-metal hybrid structures. Weld. World.

[38] Li X., Xu D., Gong N., Xu Z., Wang L., Dong W. (2019). Improving the strength of injection molded aluminum/polyphenylene sulfide lap joints dependence on surface microstructure and composition. Mater. Des..

[39] Li X., Liu F., Gong N., Yang C., Wang B. (2018). Surface topography induced high injection joining strength of polymer-metal composite and fracture mechanism. Compos. Struct..

[40] Amancio-Filho S.T., Dos Santos J.F. (2009). Joining of polymers and polymer-metal hybrid structures: Recent developments and trends. Polym. Eng. Sci..

[41] Huang Y., Meng X., Xie Y., Wan L., Lv Z., Cao J., Feng J. (2018). Friction stir welding/processing of polymers and polymer matrix composites. Compos. Part A Appl. Sci. Manuf..

[42] Eshtayeh M.M., Hrairi M., Mohiuddin A.K.M. (2015). Clinching process for joining dissimilar materials: State of the art. Int. J. Adv. Manuf. Technol..

[43] Lambiase F., Balle F., Blaga L.-A., Liu F., Amancio-Filho S.T. (2021). Friction-based processes for hybrid multi-material joining. Compos. Struct..

[44] He X. (2017). Clinching for sheet materials. Sci. Technol. Adv. Mater..

[45] Zhang Y., Shan H., Li Y., Guo J., Luo Z., Ma C.Y. (2017). Joining aluminum alloy 5052 sheets via novel hybrid resistance spot clinching process. Mater. Des..

[46] Zhao L., He X.C., Lu Y. (2014). Study on Clinching of Titanium Alloy. Appl. Mech. Mater..

[47] Osten J., Söllig P., Reich M., Kalich J., Füssel U., Keßler O. (2014). Softening of High-Strength Steel for Laser Assisted Clinching. Adv. Mater. Res..

[48] Lambiase F., Paoletti A. (2018). Friction-assisted clinching of Aluminum and CFRP sheets. J. Manuf. Process..

[49] Lambiase F., di Ilio A. (2018). Joining Aluminum with Titanium alloy sheets by mechanical clinching. J. Manuf. Process..

[50] Lee C.J., Kim B.M., Kang B.S., Song W.J., Ko D.C. (2017). Improvement of joinability in a hole clinching process with aluminum alloy and carbon fiber reinforced plastic using a spring die. Compos. Struct..

[51] Lee S.H., Lee C.J., Kim B.H., Ahn M.S., Kim B.M., Ko D.C. (2014). Effect of Tool Shape on Hole Clinching for CFRP with Steel and Aluminum Alloy Sheet. Key Eng. Mater..

[52] Varis J. (2006). Economics of clinched joint compared to riveted joint and example of applying calculations to a volume product. J. Mater. Process. Technol..

[53] Pressotechnik T. https://us.tox-pressotechnik.com/.

[54] Lambiase F. (2015). Mechanical behaviour of polymer–metal hybrid joints produced by clinching using different tools. Mater. Des..

[55] Varis J. (2006). Ensuring the integrity in clinching process. J. Mater. Process. Technol..

[56] Abe Y., Mori K., Kato T. (2012). Joining of high strength steel and aluminium alloy sheets by mechanical clinching with dies for control of metal flow. J. Mater. Process. Technol..

[57] Oudjene M., Ben-Ayed L., Delamézière A., Batoz J.L. (2009). Shape optimization of clinching tools using the response surface methodology with Moving Least-Square approximation. J. Mater. Process. Technol..

[58] de Paula A.A., Aguilar M.T.P., Pertence A.E.M., Cetlin P.R. (2007). Finite element simulations of the clinch joining of metallic sheets. J. Mater. Process. Technol..

[59] Jayasekara V., Min K.H., Noh J.H., Kim M.T., Seo J.M., Lee H.Y., Hwang B.B. (2010). Rigid-plastic and elastic-plastic finite element analysis on the clinching joint process of thin metal sheets. Metals Mater. Int..

[60] Lee C.-J., Kim J.-Y., Lee S.-K., Ko D.-C., Kim B.-M. (2010). Parametric study on mechanical clinching process for joining aluminum alloy and high-strength steel sheets. J. Mech. Sci. Technol..

[61] Lambiase F., di Ilio A. (2016). Damage analysis in mechanical clinching: Experimental and numerical study. J. Mater. Process. Technol..

[62] Varis J.P., Lepistö J. (2003). A simple testing-based procedure and simulation of the clinching process using finite element analysis for establishing clinching parameters. Thin-Walled Struct..

[63] Lee C.-J., Kim J.-Y., Lee S.-K., Ko D.-C., Kim B.-M. (2010). Design of mechanical clinching tools for joining of aluminium alloy sheets. Mater. Des..

[64] Coppieters S., Lava P., Baes S., Sol H., van Houtte P., Debruyne D. (2012). Analytical method to predict the pull-out strength of clinched connections. Thin-Walled Struct..

[65] Zhao S.D., Xu F., Guo J.H., Han X.L. (2014). Experimental and numerical research for the failure behavior of the clinched joint using modified Rousselier model. J. Mater. Process. Technol..

[66] Song Y., Yang L., Zhu G., Hua L., Liu R. (2019). Numerical and experimental study on failure behavior of steel-aluminium mechanical clinched joints under multiple test conditions. Int. J. Lightweight Mater. Manuf..

[67] Mucha J. (2017). Clinching technology in the automotive industry. Arch. Automot. Eng..

[68] Babalo V., Fazli A., Soltanpour M. (2018). Electro-Hydraulic Clinching: A novel high speed joining process. J. Manuf. Process..

[69] Lambiase F. (2012). Influence of process parameters in mechanical clinching with extensible dies. Int. J. Adv. Manuf. Technol..

[70] Neugebauer R., Kraus C., Dietrich S. (2008). Advances in mechanical joining of magnesium. CIRP Ann..

[71] Busse S., Merklein M., Roll K., Ruther M., Zürn M. (2010). Development of a mechanical joining process for automotive body-in-white production. Int. J. Mater. Form..

[72] Lee C.-J., Lee J.-M., Ryu H.-Y., Lee K.-H., Kim B.-M., Ko D.-C. (2014). Design of hole-clinching process for joining of dissimilar materials—Al6061-T4 alloy with DP780 steel, hot-pressed 22MnB5 steel, and carbon fiber reinforced plastic. J. Mater. Process. Technol..

[73] Wen T., Wang H., Yang C., Liu L.T. (2014). On a reshaping method of clinched joints to reduce the protrusion height. Int. J. Adv. Manuf. Technol..

[74] Shi C., Yi R., Chen C., Peng H., Ran X., Zhao S. (2020). Forming mechanism of the repairing process on clinched joint. J. Manuf. Process..

[75] He X., Pearson I., Young K. (2008). Self-pierce riveting for sheet materials: State of the art. J. Mater. Process. Technol..

[76] B. Group https://www.boellhoff.com.

[77] Fastening S.E. https://www.stanleyengineeredfastening.com.

[78] Li D., Han L., Thornton M., Shergold M. (2012). Influence of edge distance on quality and static behaviour of self-piercing riveted aluminium joints. Mater. Des..

[79] Li D., Chrysanthou A., Patel I., Williams G. (2017). Self-piercing riveting-a review. Int. J. Adv. Manuf. Technol..

[80] Porcaro R., Hanssen A.G., Langseth M., Aalberg A. (2006). Self-piercing riveting process: An experimental and numerical investigation. J. Mater. Process. Technol..

[81] Xie Z., Yan W., Yu C., Mu T., Song L. (2018). Tensile capacity of self-piercing rivet connections in thin-walled steel structures. J. Constr. Steel Res..

[82] Hou W., Mangialardi E., Hu S.J., Wang P.C., Menass R. Characterization for quality monitoring of a self-piercing riveting process. Proceedings of the Sheet Metal Welding conference XI, Sterling Hieights.

[83] Borba N.Z., Blaga L., Dos Santos J.F., Amancio-Filho S.T. (2018). Direct-Friction Riveting of polymer composite laminates for aircraft applications. Mater. Lett..

[84] Borba N.Z., Kötter B., Fiedler B., Dos Santos J.F., Amancio-Filho S.T. (2020). Mechanical integrity of friction-riveted joints for aircraft applications. Compos. Struct..

[85] Amancio-Filho S.T., Beyer M., Dos Santos J.F. (2009). Method of Connecting a Metallic Bolt to a Plastic Workpiece. U.S. Patent.

[86] Amancio-Filho S.T., Blaga L.-A. (2018). Joining of Polymer-Metal Hybrid Structures: Principles and Applications.

[87] Gagliardi F., Conte R., Ciancio C., Simeoli G., Pagliarulo V., Ambrogio G., Russo P. (2018). Joining of thermoplastic structures by Friction Riveting: A mechanical and a microstructural investigation on pure and glass reinforced polyamide sheets. Compos. Struct..

[88] Hynes N.R.J., Vignesh N.J., Velu P.S. (2020). Low-speed friction riveting: A new method for joining polymer/metal hybrid structures for aerospace applications. J. Braz. Soc. Mech. Sci. Eng..

[89] Okada T., Uchida S., Nakata K. (2014). Direct Joining of Aluminum Alloy and Plastic Sheets by Friction Lap Processing. Mater. Sci. Forum.

[90] Nagatsuka K., Yoshida S., Tsuchiya A., Nakata K. (2015). Direct joining of carbon-fiber–reinforced plastic to an aluminum alloy using friction lap joining. Compos. Part B Eng..

[91] Liu F.C., Nakata K., Liao J., Hirota S., Fukui H. (2014). Reducing bubbles in friction lap welded joint of magnesium alloy and polyamide. Sci. Technol. Weld. Join..

[92] Lambiase F., Grossi V., Paoletti A. (2020). Friction Stir Joining of CFRP laminates with amorphous polymers: Influence of processing speeds. J. Manuf. Process..

[93] Lambiase F., Grossi V., Paoletti A. (2020). Effect of tilt angle in FSW of polycarbonate sheets in butt configuration. Int. J. Adv. Manuf. Technol..

[94] Manente André N., dos Santos J.F., TAmancio-Filho S. (2019). Evaluation of Joint Formation and Mechanical Performance of the AA7075-T6/CFRP Spot Joints Produced by Frictional Heat. Materials.

[95] Lambiase F., Genna S. (2017). Laser-assisted direct joining of AISI304 stainless steel with polycarbonate sheets: Thermal analysis, mechanical characterization, and bonds morphology. Opt. Laser Technol..

[96] Liu F.C., Dong P., Lu W., Sun K. (2019). On formation of Al-O-C bonds at aluminum/polyamide joint interface. Appl. Surf. Sci..

[97] Liu F.C., Liao J., Gao Y., Nakata K. (2015). Effect of plasma electrolytic oxidation coating on joining metal to plastic. Sci. Technol. Weld. Join..

[98] Nagatsuka K., Kitagawa D., Yamaoka H., Nakata K. (2016). Friction Lap Joining of Thermoplastic Materials to Carbon Steel. ISIJ Int..

[99] Wu L.H., Nagatsuka K., Nakata K. (2018). Direct joining of oxygen-free copper and carbon-fiber-reinforced plastic by friction lap joining. J. Mater. Sci. Technol..

[100] Nagatsuka K., Xiao B., Wu L., Nakata K., Saeki S., Kitamoto Y., Iwamoto Y. (2018). Resistance spot welding of metal/carbon-fibre-reinforced plastics and applying silane coupling treatment. Sci. Technol. Weld. Join..

[101] Ageorges C., Ye L. (2001). Resistance welding of metal/thermoplastic composite joints. J. Thermoplast. Compos..

[102] Katayama S., Kawahito Y. (2008). Laser direct joining of metal and plastic. Scr. Mater..

[103] Kawahito Y., Niwa Y., Terajima T., Katayama S. (2010). Laser Direct Joining of Glassy Metal Zr55Al10Ni5Cu30 to Engineering Plastic Polyethylene Terephthalate. Mater. Trans..

[104] Farazila Y., Miyashita Y., Hua W., Mutoh Y., Otsuka Y. (2011). YAG Laser Spot Welding of PET and Metallic Materials. J. Laser Micro Nanoen..

[105] Wahba M., Kawahito Y., Katayama S. (2011). Laser direct joining of AZ91D thixomolded Mg alloy and amorphous polyethylene terephthalate. J. Mater. Process. Technol..

[106] Jung K.W., Kawahito Y., Katayama S. (2011). Laser direct joining of carbon fibre reinforced plastic to stainless steel. Sci. Technol. Weld. Join..

[107] Jung K.W., Kawahito Y., Takahashi M., Katayama S. (2013). Laser direct joining of carbon fiber reinforced plastic to zinc-coated steel. Mater. Des..

[108] Yusof F., Yukio M., Yoshiharu M., Shukor M.H.A. (2012). Effect of anodizing on pulsed Nd:YAG laser joining of polyethylene terephthalate (PET) and aluminium alloy (A5052). Mater. Des..

[109] Hussein F.I., Akman E., Oztoprak B.G., Gunes M., Gundogdu O., Kacar E., Hajim K.I., Demir A. (2013). Evaluation of PMMA joining to stainless steel 304 using pulsed Nd:YAG laser (vol 49, pg 143, 2013). Opt. Laser Technol..

[110] Hussein F.I., Salloomi K.N., Akman E., Hajim K.I., Demir A. (2017). Finite element thermal analysis for PMMA/st.st.304 laser direct joining. Opt. Laser Technol..

[111] Zhang Z., Shan J.G., Tan X.H. (2018). Evaluation of the CFRP grafting and its influence on the laser joining CFRP to aluminum alloy. J. Adhes. Sci. Technol..

[112] Ai Y.W., Zheng K., Shin Y.C., Wu B.X. (2018). Analysis of weld geometry and liquid flow in laser transmission welding between polyethylene terephthalate (PET) and Ti6A14V based on numerical simulation. Opt. Laser Technol..

[113] Chan C.W., Smith G.C. (2016). Fibre laser joining of highly dissimilar materials: Commercially pure Ti and PET hybrid joint for medical device applications. Mater. Des..

[114] Jung D.J., Cheon J., Na S.J. (2016). Effect of surface pre-oxidation on laser assisted joining of acrylonitrile butadiene styrene (ABS) and zinc-coated steel. Mater. Des..

[115] Yusof F., Miyashita Y., Seo N., Mutoh Y., Moshwan R. (2012). Utilising friction spot joining for dissimilar joint between aluminium alloy (A5052) and polyethylene terephthalate. Sci. Technol. Weld. Join..

[116] Esteves J.V., Goushegir S.M., Dos Santos J.F., Canto L.B., Hage E., Amancio S.T. (2015). Friction spot joining of aluminum AA6181-T4 and carbon fiber-reinforced poly(phenylene sulfide): Effects of process parameters on the microstructure and mechanical strength. Mater. Des..

[117] Balle F., Wagner G., Eifler D. (2007). Ultrasonic spot welding of aluminum sheet/carbon fiber reinforced polymer-joints. Mater. Werkst.

[118] Balle F., Eifler D. (2012). Statistical test planning for ultrasonic welding of dissimilar materials using the example of aluminum-carbon fiber reinforced polymers (CFRP) joints. Mater. Werkst..

[119] Lambiase F., Paoletti A. (2018). Mechanical behavior of AA5053/polyetheretherketone (PEEK) made by Friction Assisted Joining. Compos. Struct..

[120] Lambiase F., Paoletti A., Grossi V., di Ilio A. (2017). Friction assisted joining of aluminum and PVC sheets. J. Manuf. Process..

[121] Lambiase F., Genna S. (2020). Homogenization of temperature distribution at metal-polymer interface during Laser Direct Joining. Opt. Laser Technol..

[122] Lambiase F., Grossi V., Paoletti A. (2020). Machine learning applied for process design of hybrid metal-polymer joints. J. Manuf. Process..

[123] Lee C.-J., Shen G., Kim B.-M., Lambiase F., Ko D.-C. (2018). Analysis of Failure-Mode Dependent Joint Strength in Hole Clinching from the Aspects of Geometrical Interlocking Parameters. Metals.

[124] Liu Y., Zhuang W. (2019). Self-piercing riveted-bonded hybrid joining of carbon fibre reinforced polymers and aluminium alloy sheets. Thin-Walled Struct..

[125] Abibe A.B., Sônego M., Dos Santos J.F., Canto L.B., Amancio-Filho S.T. (2016). On the feasibility of a friction-based staking joining method for polymer–metal hybrid structures. Mater. Des..

[126] Zinn C., Bobbert M., Dammann C., Wang Z., Tröster T., Mahnken R., Meschut G., Schaper M. (2018). Shear strength and failure behaviour of laser nano-structured and conventionally pre-treated interfaces in intrinsically manufactured CFRP-steel hybrids. Compos. Part B Eng..

[127] Meng X., Huang Y., Cao J., Shen J., Dos Santos J.F. (2021). Recent progress on control strategies for inherent issues in friction stir welding. Prog. Mater. Sci..

[128] Lambiase F., Grossi V., Paoletti A. (2021). Defects formation during Friction Assisted Joining of metals and semi crystalline polymers. J. Manuf. Process..

[129] André N.M., Goushegir S.M., Dos Santos J.F., Canto L.B., Amancio-Filho S.T. (2016). Friction Spot Joining of aluminum alloy 2024-T3 and carbon-fiber-reinforced poly(phenylene sulfide) laminate with additional PPS film interlayer: Microstructure, mechanical strength and failure mechanisms. Compos. Part B Eng..

[130] André N.M., Goushegir S.M., Dos Santos J.F., Canto L.B., Amancio-Filho S.T. (2016). Influência da Espessura do Filme Polimérico Intermediário na Resistência Mecânica de Juntas Híbridas de Alumínio 2024-T3 e CF-PPS Produzidas por União Pontual por Fricção. Soldag. Inspeção.

[131] André N.M., Goushegir S.M., Scharnagl N., Dos Santos J.F., Canto L.B., Amancio-Filho S.T. (2018). Composite surface pre-treatments: Improvement on adhesion mechanisms and mechanical performance of metal–composite friction spot joints with additional film interlayer. J. Adhes..

